# Youth Soccer Development After a Forced Training Interruption: A Retrospective Analysis of Prepubertal Players

**DOI:** 10.3390/sports13120435

**Published:** 2025-12-04

**Authors:** Federico Abate Daga, Italo Sannicandro, Alice Tanturli, Samuel Agostino

**Affiliations:** 1 Department of Clinical and Biological Sciences, University of Turin, 10124 Turin, Italy; 2 Department of Experimental and Clinical Medicine, University of Foggia, 71122 Foggia, Italy; 3 Department of Experimental and Clinical Medicine, University of Florence, 50121 Florence, Italy; 4 Department of Medical Sciences, University of Turin, 10124 Turin, Italy

**Keywords:** youth soccer, training interruption, motor performance, technical abilities, children

## Abstract

This retrospective, non-interventional study investigated the impact of a forced training interruption, such as the COVID-19 nationwide lockdown, on the physical efficiency, performance, and technical-agility skills of prepubertal soccer players by comparing pre- and post-interruption cohorts within the same youth academy and at the same chronological age. Anonymised data collected across multiple competitive seasons included anthropometric measures and motor performance tests: Standing Long Jump, Shuttle Run, Shuttle Dribble, and Mini Cooper. Between-group differences were analysed using Mann–Whitney U tests, with *p* < 0.05 as the level of significance. The post-training interruption cohort showed significantly higher values in the Standing Long Jump (+11.2%, *p* < 0.001) and the Shuttle Run (+8.0%, *p* = 0.011), indicating improved explosive power and agility-speed. Conversely, performance on the Shuttle Dribble test declined by 13.4% (*p* < 0.001), while Mini Cooper results and BMI did not differ significantly. These findings suggest that modifications to compulsory training and children’s natural adaptability may have enhanced physical abilities, whereas the lack of contextual and interactive practice negatively affected technical-agility skills. Coaches should incorporate alternative workouts to maintain motor performance and emphasise ball-related and decision-making drills within ecological and dynamic environments to maximise developmental outcomes. This study offers new insights into the adaptive responses of young athletes and provides practical lessons for future youth soccer development, particularly during periods of forced training interruption.

## 1. Introduction

Soccer is one of the most widely practised sports worldwide, characterised by a continuous alternation of high-intensity efforts, rapid accelerations, and technical–tactical decisions. It requires integrating physical, technical, cognitive, and emotional components that together determine performance [1]. During the prepubertal stage, soccer training focuses on consolidating the basic elements of movement and coordination that underpin sport-specific skills. Therefore, at this stage, soccer training aims to progressively enhance fundamental movement skills, coordination, agility, and ball control [2,3]. Sessions are centred on multidirectional movements, ball handling, and simple game situations that stimulate decision-making and spatial awareness. This stage is considered a transitional phase in which general motor abilities, such as running, jumping, and changing direction, are gradually integrated with the technical foundations of soccer, including passing, dribbling, and ball control [4,5,6]. Thus, maintaining training continuity during these years is essential, as it allows children to progressively refine their motor repertoire and adapt to the game’s physical and perceptual demands. Interruptions or irregular practice can hinder the acquisition of complex skills, leading to reduced confidence and fluency in movement execution, with potential consequences for future development and participation in sports [7]. At the U9 stage, children are consistently prepubertal and follow one of the first structured training cycles within youth soccer academies. This category represents a highly homogeneous developmental window characterised by limited hormonal variability and high neuromuscular plasticity, making U9 players particularly suitable for observing training-related adaptations under constrained conditions [8].

Unfortunately, the COVID-19 pandemic led to an unprecedented global interruption of sports activities. Lockdowns and social distancing measures led to the closure of sports facilities, the suspension of competitions, and a shift to home-based or remote training programmes. For several months, children and adolescents were deprived of structured practice, social play, and outdoor movement opportunities [9,10,11]. This sudden and prolonged inactivity raised concerns about its potential effects on both physical fitness and motor development [12,13]. In adult and professional soccer, multiple studies have documented reductions in performance parameters and, consequently, in match intensity following the return to play after lockdown [14,15]. Players covered shorter total distances and performed fewer high-intensity and sprinting actions during official matches, suggesting that the period of isolation led to measurable detraining effects [16,17]. Furthermore, the reduction in match intensity was primarily attributed to the complete suspension of collective training, the absence of a competitive rhythm, and the limited ability to reproduce game-like stimuli during home-based conditioning [18,19,20].

These findings contributed to the widespread belief that similar declines must have occurred in youth players as well. However, this assumption has rarely been supported by scientific evidence. While the physiological mechanisms of detraining are widely described in adults, children differ substantially in their growth patterns, adaptability, and training environment. Youth athletes exhibit faster neuromuscular recovery, greater motor adaptability, and a lower susceptibility to loss of physical capacity during periods of inactivity, which may explain why their responses differ from those of professional adults [8]. The young athlete’s capacity for recovery and motor relearning, as well as the specific content and objectives of youth training sessions, may yield outcomes different from those of professional players [21]. Children exhibit faster recovery, greater motor adaptability, and a lower risk of losing physical capacities during periods of inactivity. Their ongoing growth, neuromuscular plasticity, and different training demands make the mechanisms of detraining more variable and often less severe. In some cases, motor skills and general fitness can be maintained or quickly regained after short interruptions [22,23]. Furthermore, prepubertal players, who are less subject to hormonal and morphological variability than adolescents, provide an ideal model for isolating the behavioural and environmental effects of a forced training interruption on performance.

However, the pandemic restricted children’s mobility and sports participation and exacerbated disparities in motor development and access to movement opportunities, primarily influenced by living environments and socioeconomic status. Nevertheless, limited information is available on the impact of long-term interruption on motor skills and specific soccer abilities in prepubertal soccer players. Therefore, this study aimed to investigate the effects of a COVID-19-related training interruption on the motor and technical performance of U9 soccer players by comparing U9 player cohorts assessed in different seasons before and after the forced stop. Because children naturally advance through the club’s age-group pathway, each season involved an entirely new group of U9 players rather than the same individuals followed longitudinally. The specific goal was to describe developmental patterns that emerged under constrained training conditions rather than establish causal effects. Specifically, we aimed to investigate whether training interruptions impacted children’s physical efficiency and soccer-specific skills.

## 2. Materials and Methods

### 2.1. Study Design and Participants

This retrospective, non-interventional study analysed performance data collected over multiple competitive seasons from players belonging to the same youth soccer academy. We received and processed data from the 2017–2018 season to the 2023–2024 season. In each season, we included the U9 team corresponding to that specific year, meaning that every season involved a different group of players according to the club’s age-category structure. As a result, the dataset consists of cross-sectional cohorts rather than a longitudinal follow-up of the same athletes. Players were eligible for inclusion only if they had attended at least 75% of the scheduled training sessions in the four weeks preceding the testing session, as verified through the club’s internal attendance registry. A total of 147 male U9 players were included in the analysis. All participants were enrolled in a high-quality federation-certified soccer school officially affiliated with the Italian Football Federation (FIGC). The sample was divided into two cohorts: a pre-interruption group (*n* = 60; seasons 2017–2018 and 2018–2019) and a post-interruption group (*n* = 87; seasons 2021–2022, 2022–2023, and 2023–2024). These specific seasons were selected to avoid deviating excessively from the period directly affected by the forced interruption. The two intermediate seasons (2019–2020 and 2020–2021) were heavily disrupted by lockdown measures and irregular training calendars. According to the club’s official declaration, all players followed the same long-term training curriculum and were supervised by the same technical staff, ensuring consistency in methodology and session structure year after year. Furthermore, all testing sessions were conducted by the same technical staff at the academy’s training centre in the last month of the season (May), under standardised environmental conditions (sunny, warm days) on the same synthetic playing surface during a regular training session. Only players whose training attendance was formally certified and who had completed the entire battery of motor tests during their respective seasons were considered eligible for inclusion in the present study. No specific interventions were introduced for research purposes.

### 2.2. Assessments and Procedures

**Figure 1:**
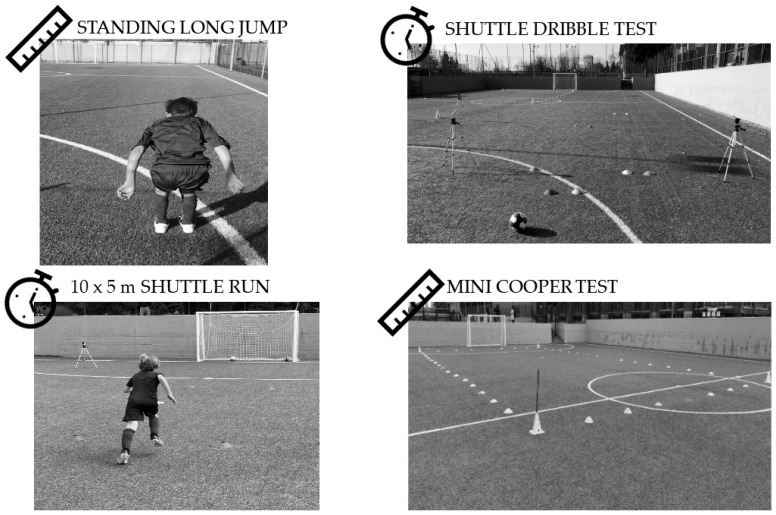
Field setups of the four tests included in the study: Standing Long Jump (**top left**), 10 × 5 m Shuttle Run (**bottom left**), Shuttle Dribble Test (**top right**), and Mini Cooper Test (**bottom right**).

From the club-provided database, we noted that a standardised battery of anthropometric and field assessments was routinely used to monitor players’ development. According to club documentation, all evaluations were carried out by the technical staff in the same order and following the same procedures each season, with less fatiguing tests performed first. Finally, no data were available regarding the pubertal status; however, all participants were aged 8–9 years. Although before puberty, age is generally considered a reliable proxy for prepubertal status in this category, the absence of a direct maturation indicator is a methodological limitation acknowledged in the present study. Therefore, they were considered prepubertal following the standard classification. Furthermore, according to the club’s procedures, anthropometrics and performance tests were administered as follows:

- Anthropometric measurements were collected before the physical tests in a dedicated room within the club facilities, where players entered individually to be measured under standardised conditions. Body mass was measured to the nearest 0.1 kg using a Rowenta BS1060 scale (Offenbach am Main, Germany), with players wearing training apparel but without shoes or shin guards. Standing height was measured with a wall-mounted stadiometer with a precision of 0.01 m and a range of 60–210 cm (Lanzoni D01602H, Bologna, Italy). Body mass index (BMI) was then calculated as weight divided by height squared (kg/m^2^) using a Microsoft Excel spreadsheet.
- Standing Long Jump (SLJ): assessed lower-limb explosive power. Players stood with both feet behind a marked take-off line and performed a two-footed jump as far forward as possible. Each participant completed three attempts, and the best performance was retained for analysis. The jump distance was measured from the take-off line to the nearest point of landing, typically the heels, by projecting a straight line backwards from the athlete’s heels to the take-off line (Figure 1).
- Shuttle Dribble Test: assessed technical-agility performance. Players completed a shuttle run while leading the ball, performing 180° changes of direction at 5, 6, 9, and 10 m within a 2 m lane. As described by Huijgen et al. [24], players dribbled 5 m, executed a 180° change of direction, dribbled 6 m back toward the start, performed another 180° change of direction, dribbled 10 m, executed a 180° change of direction, and finally dribbled 9 m back to the start/finish line. Markers were placed at each turning point, and timing gates (Microgate Witty, Bolzano, Italy) were positioned only at the start/finish line. Two trials were allowed, and the fastest time was recorded. According to the club’s information, all players completed a familiarisation trial before the official measurements were taken, ensuring that each participant understood the procedures and the execution of the tests (Figure 1).
- 10 × 5 m Shuttle Run: evaluated agility and repeated sprint ability. Each participant completed 10 times 5 m sprints with 180° changes of direction, timed manually with a professional stopwatch (Casio HS-3V-1RET, Tokyo, Japan). The test started upon the coach’s verbal “go” signal, which simultaneously activated the stopwatch, and ended when the player completed all shuttle segments. Although timing gates were available, the Shuttle Run was manually timed for both practical and methodological reasons. Practically, the test was administered after the Shuttle Dribble Test, for which the timing gates were already configured at the start/finish line, making manual timing the most efficient option. Methodologically, the Shuttle Run is part of the EUROFIT test battery for children, which recommends using a handheld stopwatch due to its practicality, widespread applicability, and established normative data collected through manual timing [25]. In contrast, the Shuttle Dribble Test, according to its original protocol, requires electronic timing gates to ensure accuracy during ball-dribbling trials [24] (Figure 1).
- Mini Cooper Test: evaluated aerobic endurance. The Mini Cooper Test used in this study corresponded to the 6 min version commonly applied in youth field testing [26]. Players ran continuously for six minutes around a 9 × 18 m rectangular course marked by cones at each corner, and the total distance covered was recorded. Children were permitted to walk whenever they could no longer run. According to the club’s guidelines, the trainer stands near the starting cone. Each time a player crossed the starting line, the coach recorded one completed lap. At the end of the six minutes, the coach signalled the cessation of the test with a whistle, and the children were instructed to stop immediately at their current position. The total distance covered was then derived by summing the number of fully completed laps and the additional portion of the final lap, calculated using the known lengths of the circuit sides (18 m for the long side and 9 m for the short side) (Figure 1).

A minimum of three minutes of passive recovery was ensured between tests to prevent fatigue interference.

### 2.3. Statistical Analysis

Data were checked for normality using the Shapiro–Wilk test. Since several variables did not meet the normality assumption, Mann–Whitney U tests were applied to compare the pre-lockdown and post-lockdown cohorts. The decision to use non-parametric tests was therefore based on the non-normal distribution observed in multiple variables, despite the relatively large sample size. Results are expressed as mean ± standard deviation (SD), and the level of significance was set at *p* < 0.05. All analyses were performed using JASP software (version 0.18.3; University of Amsterdam, Amsterdam, The Netherlands). To verify the robustness of the between-group comparisons, additional linear models were conducted, including weight, height and BMI as covariates. Results were consistent with those from the Mann–Whitney U test.

### 2.4. Ethical Considerations

This study involved a retrospective analysis of anonymised performance data collected from youth soccer players during routine athletic assessments. No personal or identifiable information was used, and no intervention or direct contact with participants occurred. In accordance with national and institutional ethical standards, formal ethics committee approval was not required for this type of study.

## 3. Results

### Descriptive and Comparative Analysis

**Figure 2:**
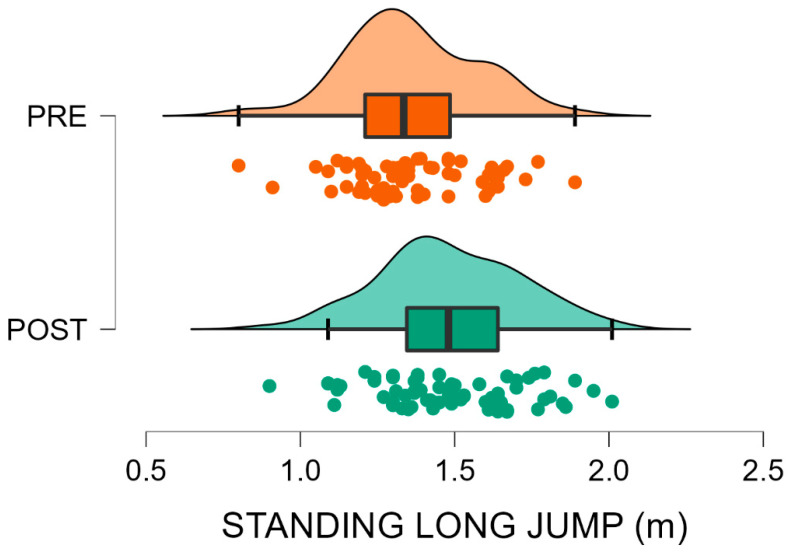
Standing Long Jump performance in pre- and post-interruption cohorts. Standing long jump distance before (PRE, orange) and after (POST, green) the forced interruption. Violin plots depict the data distribution; boxes represent mean and SD, whiskers indicate the data range, and circles show individual participant values. Post-interruption players showed an 11.2% better result (*p* < 0.001).

**Figure 3:**
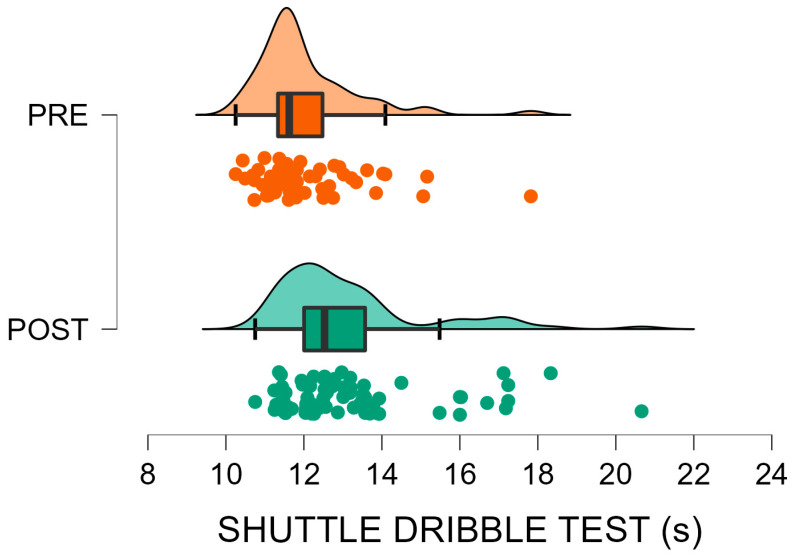
Shuttle dribble test before (PRE, orange) and after (POST, green) the forced interruption. Violin plots depict the data distribution; boxes represent mean and SD, whiskers indicate the data range, and circles show individual participant values Performance declined by 13.4% (*p* < 0.001), indicating worse results in technical agility and proficiency for the post interruption group.

**Figure 4:**
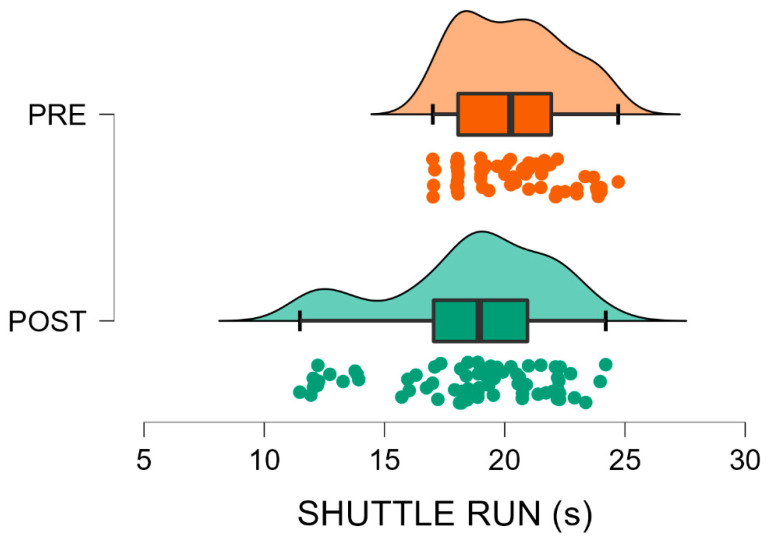
Shuttle run test before (PRE, orange) and after (POST, green) the forced interruption. Violin plots depict the data distribution; boxes represent mean and SD, whiskers indicate the data range, and circles show individual participant values Post-interruption, players showed better results, approximately 8% higher (*p* = 0.011).

**Figure 5:**
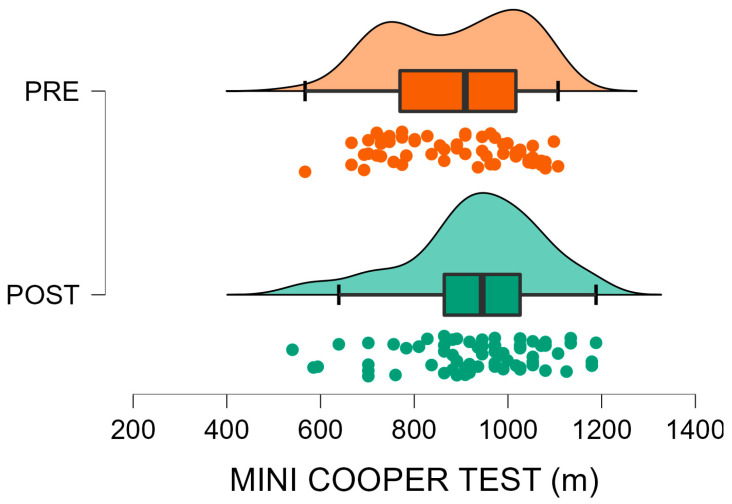
Mini cooper test before (PRE, orange) and after (POST, green) the forced interruption. Violin plots depict the data distribution; boxes represent mean and SD, whiskers indicate the data range, and circles show individual participant values. No significant difference between pre- and post-interruption groups (*p* = 0.176).

Data from 147 U9 soccer players were analysed, comparing pre (*n* = 60) and post-interruption (*n* = 87) cohorts. Descriptive statistics and Mann–Whitney U test outcomes are presented in Table 1. Post-interruption players showed significantly higher body mass (+9.3%, *p* = 0.003) and height (+2.0%, *p* = 0.003) compared with the pre-interruption cohort, while BMI did not differ significantly (*p* = 0.108). For performance tests, significantly better results were observed in the Standing Long Jump (+11.2%, *p* < 0.001) and Shuttle Run (−8.0%, *p* = 0.011) in favour of the post-interruption cohort, indicating enhanced lower-limb explosive power and running agility-speed, respectively. Conversely, performance on the Shuttle Dribble Test deteriorated (+13.4% slower time, *p* < 0.001) in the same group, suggesting a reduction in technical agility. No significant difference was found in the Mini Cooper Test distance (*p* = 0.176), indicating stable aerobic endurance between cohorts. Finally, post-interruption players demonstrated better results in tests emphasising physical power and speed, while ball-related agility appeared negatively affected (Figure 2, Figure 3, Figure 4 and Figure 5).

Figure 2, Figure 3, Figure 4 and Figure 5 visually summarise the comparative trends observed between the two cohorts. In particular, Figure 1 highlights substantially better results in explosive power, while Figure 3 shows a similar positive trend in agility and speed. Conversely, Figure 2 clearly illustrates the post-interruption decline in technical dribbling efficiency, reinforcing the notion that contextual practice is essential for skill retention. Figure 4 indicates that aerobic endurance remained stable across cohorts, suggesting that basic cardiovascular capacity was relatively preserved despite training interruptions.

Finally, ANCOVA analyses controlling for year of birth, height, and BMI yielded results comparable to those of the initial between-group tests. The post-interruption cohort maintained significantly higher SLJ and shuttle-run performance, while differences in shuttle-dribble time remained unfavourable for the post-interruption group (all *p* < 0.05). Mini-Cooper performance remained non-significant (*p* = 0.176).

## 4. Discussion

The present retrospective, non-interventional study examined the impact of forced training interruptions on motor performance in prepubertal soccer players by comparing cohorts tested before and after the pandemic. First, the anthropometric differences observed between cohorts likely reflect natural inter-individual variability in somatic growth among 8–9-year-old children. Notably, BMI did not differ significantly between groups, indicating that height and body mass increased proportionally and were not influenced by the interruption itself [27]. The main findings reveal a differentiated effect of the interruption period on children’s physical efficiency and performance. Contrary to the widespread assumption that a forced stop training period would universally impair performance (in adults) [14,16], the post-interruption cohort showed significantly higher scores in the Standing Long Jump and Shuttle Run, indicating enhanced explosive power and agility. In contrast, technical-agility performance, measured through the Shuttle Dribble Test, worsened significantly, while aerobic endurance, assessed via the Mini Cooper Test, remained stable. These results align with studies reporting that children’s general fitness and motor efficiency were not, or were only mildly, affected by interruptions and often recovered quickly once structured activity resumed [28,29]. Similarly, Neville et al. [30] observed that reductions in training volume did not always translate into lower physical performance, particularly in younger age groups characterised by high adaptability. Conversely, our findings diverge from research on adult and professional players, which showed marked decreases in aerobic capacity, high-intensity running, and match performance after interruption [14,16,31].

Moreover, the better results observed in lower-limb power and running agility-speed in favour of the post-interruption cohort can be interpreted in several ways. First, the structure of youth soccer training was compulsorily modified during and after the pandemic. Coaches were required to provide home training schedules [32] or reorganise sessions to ensure minimum distances, avoid contact games and exercises, and implement individualised workloads to comply with health and safety regulations. These mandatory adaptations may have unintentionally increased the focus on fundamental physical attributes such as strength, sprinting, and change of direction [33]. Moreover, children’s natural neuromuscular adaptability allows for rapid recovery and performance gains once regular training resumes. Prepubertal athletes display high responsiveness to varied stimuli and a capacity to regain physical qualities even after extended breaks [34]. Conversely, technical-agility performance showed the opposite trend, with lower results in the post-interruption cohort. This finding is similar to that of Le Luo et al. [35], who observed that attacking players in professional football performed fewer successful dribbles, passes, and one-on-one actions after the forced training interruption. These declines were linked to the interruption of regular match play and the reduced opportunities for decision-making, spatial awareness, and perceptual–motor interaction. Although our participants were much younger, the exact mechanisms may have influenced their Shuttle Dribble performance. Ball control at high speed and change of direction rely on repeated practice in realistic game situations, where timing, perception, and coordination are constantly challenged [5,36]. During home-based or distanced training, these conditions were largely missing, which likely limited the development of technical-agility skills. Thus, from an ecological-dynamic perspective, the constrained environment of home-based training reduced affordances and perceptual variability, which are essential for the acquisition of complex soccer skills [37]. This suggests that, even at an early age, technical abilities are highly sensitive to the training environment and require regular exposure to game-based practice to recover once normal activity resumes fully [5,38]. The prolonged absence of cooperative play may have limited perceptual–cognitive coupling, a key determinant of decision-making and anticipatory behaviour in game situations. In simpler terms, children develop technical skills through continuous exposure to varied and dynamic game situations. When these environments are restricted, as during lockdown periods, the link between what players perceive and how they respond becomes less efficient, which can limit ball control and fluency during rapid changes of direction. To facilitate the recovery of these skills once training resumes, coaches may reintroduce progressive ball-handling drills, reactive 1-vs-1 situations, small-sided games, and variable-direction dribbling tasks that recreate the perceptual and decision-making demands of match play.

Furthermore, aerobic endurance did not differ significantly between cohorts. This stability suggests that, despite the interruption, children maintained a baseline level of activity sufficient to preserve cardiovascular fitness. Similar patterns have been observed in population-based studies, which report that, although total physical activity decreased during interruptions [39], light-to-moderate activity and outdoor play partially compensated for the reduction in structured sport [40]. Unlike adults, whose aerobic capacity declines rapidly with inactivity, children tend to remain active through unstructured play and spontaneous movement. This finding may also be related to physiological differences in energy metabolism: mitochondrial structure and oxidative enzyme systems are not yet fully mature in prepubertal children. Consequently, aerobic training at this age primarily enhances fundamental motor abilities and movement efficiency rather than the aerobic mechanism itself [27,41]. Moreover, the Mini Cooper Test reflects general aerobic efficiency rather than sport-specific endurance and may therefore be less sensitive to moderate fluctuations in training volume.

Finally, this study has several limitations that must be acknowledged. Its retrospective nature means that the researchers had no direct control over testing procedures or contextual variables. Although all tests were reportedly administered following the same standardised protocol by the same technical staff, unmeasured differences in motivation, maturation timing, or seasonal factors cannot be excluded. Another limitation is the absence of a direct measure of biological maturation. Although all participants were 8–9 years old and therefore expected to be prepubertal according to standard classifications, we could not assess individual maturation differences without a specific indicator such as Tanner staging. Furthermore, no information was available regarding children’s unstructured or informal physical activity during the interruption period. Activities such as outdoor play, spontaneous movement, or home-based exercise may have contributed to maintaining or enhancing physical fitness, and their absence from the dataset limits the interpretation of individual variability. However, according to the information available from the club, all children lived in similar urban contexts, suggesting broadly comparable opportunities for spontaneous play. Nonetheless, the specific ways in which each child’s activity was organised outside the academy’s training sessions remain unknown.

Despite these constraints, the study presents two notable strengths. First, it draws upon a large sample of prepubertal players assessed under real-world training conditions, providing strong ecological validity and practical relevance for youth soccer programmes. Second, the dataset spans multiple competitive seasons within the same academy, ensuring methodological consistency and enabling meaningful comparisons between pre- and post-interruption cohorts. Together, these elements offer a robust, contextually grounded picture of youth soccer development during and after a forced training interruption. Future studies should longitudinally track cohorts across multiple developmental stages to determine whether early motor adaptations persist or converge over time.

## 5. Conclusions

This retrospective study showed that the forced training interruption did not uniformly reduce the physical performance of prepubertal soccer players. The post-interruption cohort displayed higher explosive power and agility speed, but reduced technical agility skills, while aerobic endurance remained stable. These findings suggest that modifications to compulsory training and children’s natural adaptability may favour the recovery of physical attributes. In contrast, the lack of contextual and interactive practice limited skill-based performance. The results underscore the importance of incorporating alternative workouts, such as pure physical development sessions, to further enhance players’ motor and physical performance. At the same time, exposure to ball-related and decision-making drills should be strengthened within ecological and dynamic environments to maximise transfer and overall developmental effects. From a practical standpoint, these findings indicate that coaches should prioritise the early reintroduction of game-like and perceptual–motor tasks when normal training resumes, including small-sided games, reactive 1-vs-1 situations, and variable-direction dribbling circuits that recreate the decision-making demands of match play. Ultimately, this study provides meaningful insights into the adaptive responses of young athletes to prolonged training interruptions, offering evidence-based guidance for the future design and optimisation of youth soccer development programmes post-interruption. These findings highlight the need for youth coaches to balance physical conditioning with contextualised skill exposure, ensuring that motor learning remains robust even under constrained circumstances such as health emergencies. Implementing these targeted strategies may help accelerate the recovery of technical-agility skills and support more complete development during future return-to-play phases.

## Figures and Tables

**Table 1:** Descriptive statistics and between-group comparisons (pre- vs. post-interruption cohorts). Values are expressed as mean ± standard deviation (SD). Mann–Whitney U test was applied. Significant differences are indicated with an asterisk (*) (*p* < 0.05). Δ% calculated relative to pre-interruption mean.

Variable	Pre-Interruption (Mean ± SD)	Post-Interruption (Mean ± SD)	Δ%	*p*-Value
Body mass (kg)	28.81 ± 4.40	31.51 ± 6.16	+9.3%	0.003 *
Height (m)	1.315 ± 0.057	1.341 ± 0.053	+2.0%	0.003 *
BMI (kg/m^2^)	16.61 ± 1.95	17.48 ± 2.89	+5.3%	0.108
Standing Long Jump (m)	1.36 ± 0.21	1.52 ± 0.24	+11.2%	<0.001 *
Shuttle Dribble Test (s)	12.03 ± 1.30	13.64 ± 2.74	−13.4% (slower)	<0.001 *
Shuttle Run (s)	20.33 ± 2.15	18.71 ± 3.12	+8.0% (faster)	0.011 *
Mini Cooper Test (m)	889.2 ± 140.9	922.8 ± 148.4	+3.8%	0.176

## Data Availability

The data presented in this study are available on reasonable request from the corresponding author. Due to restrictions imposed by the club owner and in compliance with Italian data protection regulations (Legislative Decree No. 196/2003, as amended by Legislative Decree No. 101/2018, which implements EU Regulation 2016/679—General Data Protection Regulation), the datasets are not publicly available.
